# Calibrated delivery drape versus indirect gravimetric technique for the measurement of blood loss after delivery: a randomized trial

**DOI:** 10.1186/1471-2393-14-276

**Published:** 2014-08-15

**Authors:** Shubha Ambardekar, Tara Shochet, Hillary Bracken, Kurus Coyaji, Beverly Winikoff

**Affiliations:** Department of Obstetrics & Gynecology, K.E.M. Hospital, Pune, India; Gynuity Health Projects, New York, NY USA

**Keywords:** Postpartum blood loss, Postpartum blood measurement

## Abstract

**Background:**

Trials of interventions for PPH prevention and treatment rely on different measurement methods for the quantification of blood loss and identification of PPH. This study’s objective was to compare measures of blood loss obtained from two different measurement protocols frequently used in studies.

**Methods:**

Nine hundred women presenting for vaginal delivery were randomized to a direct method (a calibrated delivery drape) or an indirect method (a shallow bedpan placed below the buttocks and weighing the collected blood and blood-soaked gauze/pads). Blood loss was measured from immediately after delivery for at least one hour or until active bleeding stopped.

**Results:**

Significantly greater mean blood loss was recorded by the direct than by the indirect measurement technique (253.9 mL and 195.3 mL, respectively; difference = 58.6 mL (95% CI: 31–86); p < 0.001). Almost twice as many women in the direct than in the indirect group measured blood loss > 500 mL (8.7% vs. 4.7%, p = 0.02).

**Conclusions:**

The study suggests a real and significant difference in blood loss measurement between these methods. Research using blood loss measurement as an endpoint needs to be interpreted taking measurement technique into consideration.

**Trial registration:**

This study has been registered at clinicaltrials.gov as NCT01885845.

## Background

Post-partum hemorrhage (PPH) is a leading cause of maternal mortality and morbidity around the world [1]. Accurate assessment of blood loss is important for conducting research on the effectiveness of different approaches to the prevention and treatment of PPH. Research has shown that whether or not blood loss is measured may influence reported PPH rates [2]. However, the way in which blood loss is measured may also impact the measurement itself. These differences in measurement techniques may have important implications for our understanding of reported PPH rates, the evaluation of prevention and treatment strategies, and estimates of prevented PPH.

Several techniques for measuring blood loss have been used in research trials of PPH prevention and treatment methods. Visual estimation has been found to be highly inaccurate with substantial under-estimation of blood loss at high volumes of loss [3, 4]. A second method employs an *indirect* technique with collection of blood and blood-soaked material into a vessel. The vessel is then weighed and an equivalent volume calculated. A systematic review conducted in 2010 identified more than 20 trials that had employed this method [5]. More recently, five studies have employed a *direct* technique that allows the provider to assess the blood loss as it accumulates in a calibrated receptacle (usually a drape) beneath the woman [6–10].

Although both direct and indirect methods have been widely used in clinical studies, no research has directly compared the two blood measurement techniques to assess if they produce comparable results. Understanding the equivalency or systematic differences of methods of measurement is critical to proper interpretation of findings in research that uses blood loss as an endpoint. This study sought to conduct such a comparison.

## Methods

**Figure 1:**
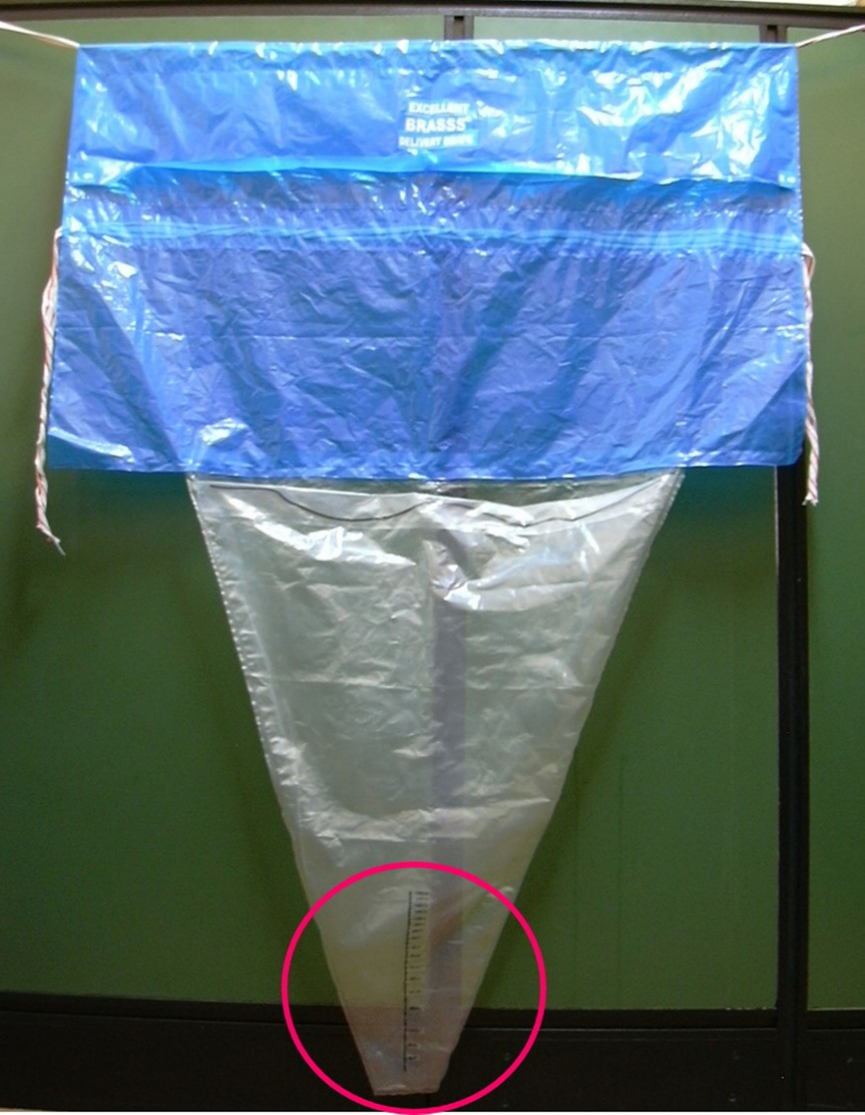
**Photo of the Excellent BRASSS-V Drape™, a blood collection drape with a calibrated collection pouch.**

This study was a randomized trial to evaluate the measurement of blood loss after delivery using two different measurement techniques: a direct method, the Excellent BRASSS-V Drape™, whereby the amount of blood is measured at the time of bleeding (see Figure 1); and an indirect method involving the weight and measurement of blood and blood-soaked materials following the cessation of bleeding. This study was purely an assessment of measurement tools and not an evaluation of the effect of the measurement technique on provider practice or blood loss interpretation. We assumed that in a large sample of normal vaginal deliveries randomized only to a difference in blood measurement techniques, the mean blood loss recorded would not be significantly different if the two methods had the same reliability.

All women aged 18 and older presenting at the study site (KEM Hospital in Pune, India) for an imminent vaginal delivery were considered eligible and were invited to join the study. Women who agreed to participate were randomized to one of the two measurement methods. Cards indicating group allocation were placed in opaque sequentially numbered envelopes, randomized in blocks of 10 via a computerized randomization sequence generated in New York by Gynuity Health Projects staff. Envelopes were opened by study staff after enrollment. All participants received the standard care provided during the third stage of labor at the study hospital. Provider actions related to the prevention or treatment of PPH (including blood loss interpretation) were as per provider preference and standard hospital practice and not dictated by the study protocol. Standard active management at KEM Hospital is 10 units oxytocin IM or IV immediately after the birth of the shoulder of the baby. Any participant who was referred for a cesarean section was withdrawn from the study post-randomization; the decision to be given a cesarean section was made independently by each provider. Enrollment continued until blood loss in 900 women was measured. The hospital received IRB approval from the KEM Research Ethics Committee and all participants signed informed consent. This study was registered retrospectively on June 19, 2013, at clinicaltrials.gov as NCT01885845.

For women randomized to the direct method (using the Excellent BRASSS-V Drape™), blood measurement began immediately after delivery and cord clamping. The calibrated delivery drape was placed under the buttocks of the woman and tied around her waist with the funnel portion hanging down between her legs. Blood loss was measured for at least one hour or, if bleeding continued after one hour, until active bleeding stopped. When active bleeding stopped, providers examined the drape and recorded the level indicated.

For women randomized to the indirect method protocol, a sheet with plastic backing was placed under the buttocks just after delivery and cord clamping. The sheet drained into a metal basin placed on a shelf below the delivery table. Blood loss was collected in the basin for at least one hour or, if bleeding continued after one hour, until active bleeding stopped. After bleeding stopped, all blood-soaked gauze pieces and mops were counted and then placed in the collection basin. The basin was placed on an electronic scale and weighed. The weight of the blood was assessed by subtracting the initial weight of the basin, gauzes and mops from the total weight of the soaked materials assuming that one gram is equivalent to 1 mL.

Hemoglobin measurements were taken at admission for delivery and at approximately twenty-four hours post-delivery. Hemoglobin was measured with an automated flow cytometric method on a coulter machine, the standard method used at KEM hospital. Study staff also recorded the amount of IV fluids, any transfusion received, time of IV removal, and time of hemoglobin assessment.

This study was powered to test the difference in mean blood loss measurement during the third stage of labor with the two techniques described above. We assumed a mean blood loss of 450 mL with the direct method and 500 mL with the indirect method, with a standard deviation of approximately 265 mL. We determined that a sample size of 900 women, 450 women in each arm, would allow us to test a mean difference of 50 mL with 80 percent power and 5 percent probability that we would incorrectly find a difference in the mean blood loss measured with each technique.

Data on demographics, active management, blood loss, and hemoglobin levels were collected by research staff and compared between the two study groups. Wilcoxon rank-sum tests, Fisher’s exact tests, and Pearson’s chi-squared tests were run as appropriate to compare frequencies and means. We also conducted linear and logistic regression. All data were entered into Statistics Package for Social Sciences (SPSS, Version 13.0, Chicago, IL). Analyses were conducted using Stata (Version 11, College Station, TX) and SPSS, Version 20.0.

## Results

**Figure 2:**
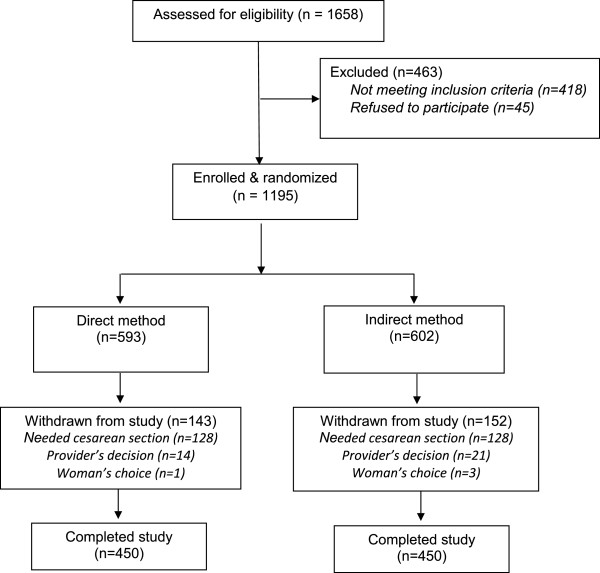
**Flowchart of study participation.**

Eleven hundred and ninety-five women were enrolled in the study between January 2006 and September 2007 (see Figure 2). Following randomization, 295 women were withdrawn from the study, mostly because of the provider’s decision to perform Caesarean section (n = 256). Other reasons for withdrawal included provider’s decision for other clinical reasons (n = 35), and woman’s choice (n = 4). The remaining 900 participants were equally divided between the two study groups. There were no significant differences in background characteristics or delivery details between the study arms (see Table 1).

**Table 1:** **Background characteristics and delivery details**

	Direct blood loss measurement	Indirect blood loss measurement	p-value ^a^
	(n = 450)	(n = 450)	
Maternal age in years: median (range)	25 (17–37)	24 (18–39)	0.58
Parity: median (range)	1 (0–4)	1 (0–4)	0.96
Gravidity: median (range)	2 (1–7)	2 (1–6)	0.69
Gestational age in weeks: median (range)	39 (28–43)	39 (28–43)	0.62
Nulliparous: % (n)	47.3 (213)	48.9 (220)	0.69
Pre-delivery hemoglobin: median (range)	11.2 (6.1–15.3)^b^	11.3 (5.5–16.3)^c^	0.27
Type of delivery: % (n)			0.90
Vaginal	91.8 (413)	92.2 (415)	
Forceps	8.2 (37)	7.8 (35)	
Delivery outcome: % (n)			> 0.99
Singleton (live birth)	99.3 (447)	99.1 (446)	
Twins (live birth)	0.2 (1)	0.4 (2)	
Triplets (live birth)	0.2 (1)	0.0 (0)	
Singleton (stillborn)	0.2 (1)	0.2 (1)	
Twins (stillborn)	0.0 (0)	0.2 (1)	
One or more complications (including vaginal or perineal tears): % (n)	1.1 (5)	2.4 (11)	0.21

^a^Calculated with the Wilcoxon rank-sum test and Fisher’s exact test.

^b^n = 422.

^c^n = 416.

All women received oxytocin as part of active management of the third stage of labor (see Table 2). Additional uterotonics included misoprostol (Direct: 11.8%; Indirect: 10.0%), carboprost (Direct: 2.0%; Indirect: 1.8%), and additional doses of oxytocin (Direct: 43.1%; Indirect: 44.7%). Almost all women in both groups (98.9%) had episiotomies performed. Very few participants required manual removal of placenta (n = 6), additional surgical intervention (n = 2), intravenous fluids (n = 10), and/or blood transfusion (n = 2). There were no significant differences between the study groups in interventions provided.

**Table 2:** **Active management and additional interventions:% (n)**

	Direct blood loss measurement	Indirect blood loss measurement	p-value ^a^
	(n = 450)	(n = 450)	
Uterotonics used for active management			
Oxytocin^b^	100.0 (450)	100.0 (450)	na
Misoprostol	0.9 (4)	1.1 (5)	> 0.99
Carboprost	0.7 (3)	0.2 (1)	0.37
Additional uterotonics for treatment			
Oxytocin	43.1 (194)	44.7 (201)	0.69
Misoprostol	11.8 (53)	10.0 (45)	0.45
Carboprost	2.0 (9)	1.8 (8)	> 0.99
Episiotomy performed	98.9 (445)	98.9 (445)	> 0.99
Manual removal of placenta performed	0.4 (2)	0.9 (4)	0.69
Other surgical intervention performed			
Internal iliac ligation	0.0 (0)	0.2 (1)	> 0.99
Resuturing of episiotomy	0.2 (1)	0.0 (0)	> 0.99
Blood transfusion given	0.2 (1)	0.2 (1)	> 0.99
Intravenous fluids given for PPH treatment	0.9 (4)	1.3 (6)	0.75

^a^Calculated with Fisher’s exact test.

^b^As per the KEM protocol, all women receive 10units of oxytocin im after delivery as a standard routine management method. If the uterus is not well contracted or there is visual excessive blood loss women receive 400 mcg of oral misoprostol or 150mcg of carboprost im.

Statistically significantly greater mean blood loss was recorded by the direct measurement method than was recorded by the indirect blood measurement technique (253.9 mL and 195.3 mL, respectively; p < 0.001) (see Table 3). The difference in mean blood loss between methods was 58.6 mL (95% CI: 31.1 – 86.1) (data not shown). In addition, almost twice as many women in the direct than in the indirect group measured a blood loss of more than 500 mL (8.7% vs. 4.7%, p = 0.02). We conducted a linear regression (of blood loss in mL) and a logistic regression (of blood loss > 500 mL), controlling for woman’s age, parity, gestational age, and pre-delivery hemoglobin level. Method of measurement retained its significant difference in both (linear regression: p < 0.001, logistic regression: p = 0.04; data not shown). Participants in both arms used the same average number of mops (0.8; p = 0.26) and the same average number of gauzes during the delivery (3.8; p = 0.93) (data not shown). The mean change in hemoglobin did not differ significantly between the study groups (direct method: −0.8, indirect method: −0.7; p = 0.19).

**Table 3:** **Blood loss in milliliters (mL)** ^**a**^ **and change in hemoglobin**

	Direct blood loss measurement	Indirect blood loss measurement	p-value ^b^
	(n = 450)	(n = 450)	
Blood loss: % (n)			< 0.001
0–100	25.8 (116)	41.1 (185)	
101–200	30.9 (139)	32.2 (145)	
201–300	19.1 (86)	11.3 (51)	
301–400	9.3 (42)	9.3 (42)	
401–500	6.2 (28)	1.3 (6)	
> 500	8.7 (39)	4.7 (21)	
Mean blood loss: mean ± SD (range)	253.9 ± 218.2 (20–1600)	195.3 ± 201.8 (20–2000)	< 0.001
Mean change in hemoglobin: mean ± SD (range)	−0.8 ± 1.4 (−7.0–3.8)^c^	−0.7 ± 1.5 (−7.2–3.2)^d^	0.19

^a^Blood loss for direct method measured in milliliters and blood loss for indirect method measured in grams (1 g = 1 mL).

^b^Calculated with Pearson’s chi-squared test and Wilcoxon rank-sum test.

^c^n = 397.

^d^n = 384.

## Discussion

This study was designed to compare two common methods for measuring postpartum blood loss in research trials. The study suggests that there may be a real and significant difference in blood loss measurement between the two methods. The mean measured blood loss was significantly greater in the direct measurement group with nothing to indicate (i.e., differences in the populations, number of mops used, or change in Hgb levels) that the measurements should have been different, implying that the measurement technique itself may be the cause of the difference in noted blood loss between the two groups.

This is the first study to present such a comparison. This trial provides new results important for the interpretation of studies using obstetric blood loss measurement as an endpoint. Studies aimed at prevention and treatment of PPH need to take into consideration the differences in measurement produced by these two techniques and interpret their findings accordingly. If using the direct approach, for example, adjustments will need to be made when comparing findings with past literature that utilized indirect techniques. In addition, meta-analyses of blood loss will need to incorporate measurement technique into their methodologies.

Rates of PPH reported in different studies could also be influenced by measurement method; studies using the drape, for example, would produce higher rates than studies using an indirect technique. In studies where the protocol requires that a woman is treated when her blood loss reaches a specific amount, often 500 mL, understanding the variation in blood loss measurement could change both treatment practices and the total number of women who receive a given intervention.

A direct comparison of the two measurement techniques would compare the assessments of the two measurement methods on the same patient. This is not feasible, however. The background characteristics of the two measurement groups suggest that the two groups were similar, and there was no reason to suspect that the mean recorded blood loss in the two groups would be different. The same research staff recorded measurement results for both techniques. Research staff used an electronic scale to record the weight of the materials in the indirect measurement group. In the direct arm, staff recorded the level indicated on the calibrated drape.

The mean blood loss found in both study arms (Direct: 253.9 mL; Indirect: 195.3 mL) was much lower than anticipated in our projected sample size calculations. The level of blood loss in this population reflects the near universal use of oxytocin prophylaxis in contrast to studies of postpartum hemorrhage conducted in community-based settings. Still, our study had sufficient power to detect a significant difference in mean blood loss between the two measurement techniques.

Some studies using the indirect technique have relied on a different formula to convert blood loss in grams to blood loss in milliliters. Althabe et al. divided the weight in grams by 1.06 (blood density in grams per milliliters) rather than 1.0 as used in this study [11]. Our calculation is conservative and if we adjusted for the weight of blood in the indirect method then the difference in blood loss measured with the two techniques would be even greater.

Different mechanisms may have contributed to the difference in mean blood loss between the two study arms. The calibrated drape used in this study starts at the 50 mL measurement and increases in increments of 50 mL to 1000 mL. Between 1000 and 2000 mL, readings are in increments of 100 mL and between 2000 and 3000 in increments of 200 mL. The less precise measures may result in heaping of measurements at 50 mL increments for cases with less than 1000 mL blood loss (especially among women with very little blood loss) and at 100 and 200 mL increments for cases with high levels of blood loss. This may have contributed to the higher measurements with the direct method when compared to the indirect method that allowed for more precise readings with the digital scale.

Blood loss was collected in the basin for at least one hour or, if bleeding continued after one hour, until active bleeding stopped. One limitation to the study is that providers were not asked to record the start and stop time for the measurement of bleeding. It is possible that measurement of bleeding in the bedpan or indirect group was curtailed as women were uncomfortable with the bedpan and blood measurement ceased after one hour. Though any additional blood measured after the cessation of active bleeding would likely be minimal and not account for the differences seen here. Additionally, there may have been losses of fluids as blood was transferred for measurement.

## Conclusions

Based on these results, we conclude that there may be an important difference in recorded blood loss between the direct and indirect measurement methods. Measurement technique must be considered both when developing research protocols that utilize blood loss measurement as an endpoint as well as when evaluating previous studies that report quantitative blood loss after delivery.
